# Familial autosomal recessive bestrophinopathy: identification of a novel variant in *BEST1* gene and the specific metabolomic profile

**DOI:** 10.1186/s12881-020-0951-3

**Published:** 2020-01-22

**Authors:** Panpan Ye, Jia Xu, Yueqiu Luo, Zhitao Su, Ke Yao

**Affiliations:** 1 Eye Center, Second Affiliated Hospital, School of Medicine, Zhejiang University, Hangzhou, China; 2 Eye Hospital, Zhejiang University, Hangzhou, China

**Keywords:** Autosomal recessive bestrophinopathy, BEST1, Mutation, Metabolomics

## Abstract

**Background:**

Autosomal recessive bestrophinopathy (ARB) is a retinal degenerative disorder caused by *BEST1* mutations with autosomal recessive inheritance. We aim to map a comprehensive genomic and metabolomic profile of a consanguineous Chinese family with ARB.

**Methods:**

Ophthalmic examinations were performed on the affected patients with ARB. The proband was screened for potential causative mutations in a panel with 256 known retinal disease genes by using target capture sequencing. The related mutation was further validated and segregated in the family members by Sanger sequencing. In silico prediction tools were used for pathogenicity assessment. A UHPLC-MS/MS metabolomic analysis was performed to explore the disease-associated metabolic feature.

**Results:**

The affected patients from this family were characterized by low vision, the presence of subretinal fluid, macular edema, and hyperopia with coincidental angle closure. DNA sequencing identified a novel missense mutation in the *BEST1* gene c.646G > A (p.Val216Ile) of the proband. Sanger sequencing further confirmed the mutation. The missense mutation was co-segregation across the pedigree and predicted to be deleterious by SIFT (0.017). The blood metabolic profiles were highly similar among all family members probably because of the same lifestyle, habitat and genomic background. However, ARB patients presented a significant deregulation of metabolites, such as citric acid, L-Threonic acid, and eicosapentaenoic acid.

**Conclusions:**

We identified a novel disease-associated variant in the *BEST1* gene as well as a disease-specific metabolic feature in familial ARB. Our findings helped improve the understanding of ARB mechanisms.

## Background

Mutations of the *BEST1* gene can cause a series of retinal degenerative diseases which are named as the “bestrophinopathies” [1]. The most common of these diseases clinically is Best vitelliform macular dystrophy (BVMD; OMIM 153700), also known as Best disease [1]. It is characterized by the deposition of bilateral yellowish yolk-like lesions in the macula [1]. BVMD is mostly inherited in an autosomal dominant type by mutations of the *BEST1* gene [1]. An allelic disease, autosomal recessive bestrophinopathy (ARB; OMIM 611809), has been first reported by Burgess R [2]. It is also caused by *BEST1* mutation with autosomal recessive inheritance [3–5]. The clinical phenotype can be different from that of BVMD. The features of ARB present with multifocal yellow subretinal deposits, subretinal fluid, macular edema, and hyperopia with coincidental angle closure. People with ARB demonstrate a decrease in vision during the first 10 years of their life.

The exact function of the *BEST1* gene remains elusive, but it is speculated to be involved in coding for an anion channel, mainly a chloride and bicarbonate channel, or be a regulator of Ca^2+^ channels in the retinal pigment epithelium (RPE) [6]. So far, more than 250 mutations have been explored in bestrophinopathies [1]. In ARB, almost half of the mutations are located between residues 312 and 315 [2]. The most common mutation for ARB is p.R15H in several families of European ethnicity and p.R255W in Chinese ethnicity [1, 7, 8]. However, the whole genomic map of ARB and the underlying mechanism remain far from being completely understood.

The metabolome affects biological and physiological processes through modulation of genetic transcription, translation, and interactions with environmental exposures [9]. Therefore, the metabolites are closely linked with genetics and also related to the phenotype [10]. Because of the rapid development in biological techniques and bioinformatics, the metabolome has been used to not only identify biomarkers for diagnosis but also provide a therapeutic strategy in diseases [11, 12]. In retinal health and disease, some groups have performed metabolomics for disease diagnosis and potential target intervention [10]. For instance, Li et al. identified plasma metabolites as biomarkers for the diagnosis and progression staging of diabetic retinopathy [13]. Laíns et al. also successfully revealed the metabolomics profile of different stages of age-related macular degeneration [14]. In the present study, we conducted a metabolomics study using blood samples in a consanguineous Chinese ARB family including three affected patients. We aimed to explore the disease-associated genomic and metabolic feature in order to better understand the etiology of ARB and provide a potential intervention strategy.

## Methods

### Clinical diagnosis

All research involved in this study adhered to the tenets of the Declaration of Helsinki and was approved by the Institutional Review Board of Second Affiliated Hospital of Zhejiang University (2019–133) (May, 2019). Informed written consent was obtained from all participating individuals in this study.

The patients were examined at Eye Center, Second Affiliated Hospital, School of Medicine of Zhejiang University, China. They underwent detailed ophthalmic evaluation, including best correct visual acuity (BCVA), slit-lamp bio-microscopy, dilated indirect ophthalmoscopy, anterior chamber (ultrasound biomicroscopy, UBM; SUOER UBM scan SW-3200), wide-field retinal imaging (Optos 200Tx, Marlborough, MA, USA), optical coherence tomography (OCT) (Heidelberg HRT II, Heidelberg, Germany), and fundus fluorescein angiography (FFA) (Heidelberg HRT II, Heidelberg, Germany) examinations.

### DNA library preparation and target sequencing

Genomic DNA of the proband and the available family members was extracted from peripheral blood by using a DNA isolation kit (Qiagen, Hilden, Germany). A pre-capture library was prepared by using Kapa LTP library prep kit (Kapa Biosystems, Wilmington, USA) and then was captured on a custom capture panel (Agilent Sureselect, USA) which containing 256 known retinal disease genes (Additional file 1: Table S1). The enriched DNA library was sequenced on Illumina Xten Analyzers (San Diego, USA) for 150 cycles per read to generate paired-end reads. An average of 172.82X in target region was achieved, and 97.86% of the target region was covered by 10x.

### Bioinformatics analysis and sanger sequencing

After the sequencing step, raw reads were aligned to the human genome reference (hg19) by using the Burrows-Wheeler Aligner (Wellcome Trust Sanger Institute, Cambridge, UK). Single-nucleotide variants (SNVs) and Insertions and Deletions (InDels) were called by Atlas-SNP2 and Atlas-Indel, respectively. SNVs and InDels were filtered against the ExAC, gnomAD, HGVD, CHARGE, 1000 Genome, and UK10K databases and the internal database of Clinbytes Inc. with an allele frequency cutoff of 0.5 and 0.1% for recessive and dominant variants, respectively. Variants were annotated using Annotate Variation (ANNOVAR). Conservation analysis of the related homologous proteins in the mutation site was performed using the UCSC Genome Browser database. In silico gene function prediction software (e.g., SIFT, PolyPhen2, and FATHMM) was used for pathogenicity assessment. The variants were further validated and segregated by Sanger sequencing from all available family members. PCR primer sets were designed via Primer3, and the products were sequenced on an ABI 3700XL Genetic Analyzer (Thermo Fisher, USA).

### UHPLC-MS/MS metabolomics analysis

The patients’ peripheral blood was collected from the available family members, and plasma was prepared for LC-MS/MS analysis, which was performed using the Vanqiush UHPLC system (Thermo Fisher) coupled with an Orbitrap Q Exactive HF-X mass spectrometer (Thermo Fisher) operating in the data-dependent acquisition mode by Novogene Co., Ltd. (Beijing, China). The detailed information has been provided previously [15]. The samples were divided into three groups according to the genotype in chr11:61725867 loci. The plasma metabolites were compared between the homozygous variation group (A/A, *n* = 2) and the wild-type group (G/G, *n* = 3), as well as between the homozygous variation group (A/A, *n* = 2) and the heterozygosity group (A/G, *n* = 2).

## Results

**Fig. 1:**
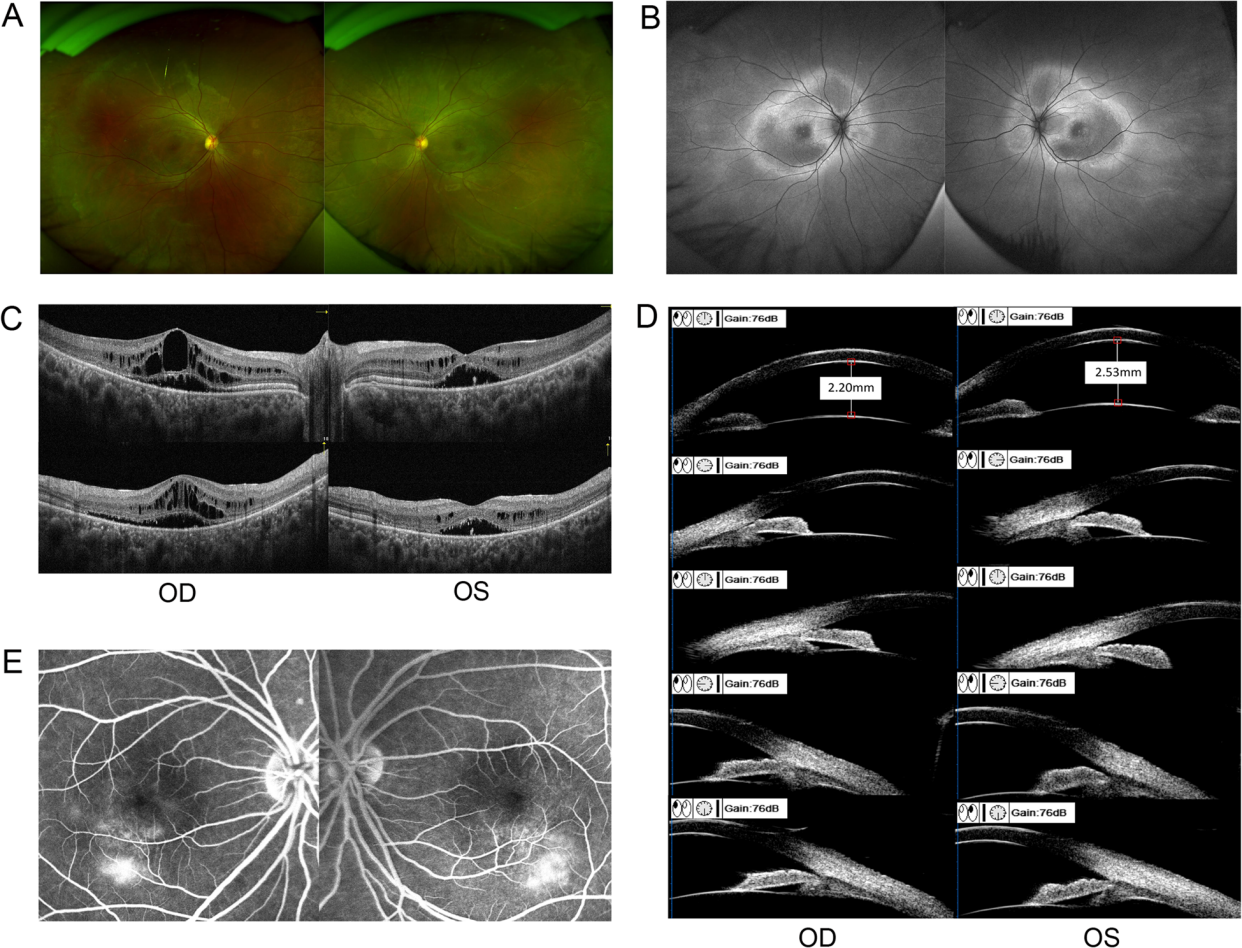
Clinical features of the ARB patients in the family. **a** Color funduscopy (CF) of the proband was normal, overall. **b** Fundus autofluorescence (FAF) image of the proband showed annular hyperfluorescence around the posterior pole. **c** OCT results of the proband revealed bilateral cystoid macular edema with subretinal fluid. **d** The affected younger sister’s ultrasound biomicroscopy (UBM) results showed a shallower anterior chamber depth, and half of the anterior chamber angles were closed. **e** The affected younger sister’s fundus fluorescence angiography (FFA) revealed mild fluorescence leakage beneath the macula

It is a consanguineous Chinese family with Han Chinese ancestry. The proband is a 34-year-old woman with progressive vision loss for over 20 years. Her BCVA was 0.15 in the right eye and 0.1 in the left eye. Color funduscopy (CF) was normal, overall (Fig. 1a). The fundus autofluorescence (FAF) image showed annular hyperfluorescence around the posterior pole (Fig. 1b). OCT results revealed cystoid macular edema with subretinal fluid (Fig. 1c). The proband has an affected younger sister aged 32, with 20 years of progressive vision loss. Her BCVA was 0.1 in both eyes. Her clinical findings in CF, FAF, and OCT were similar to those of the proband. In addition, she had a shallower anterior chamber depth, and UBM showed that half of the anterior chamber angles were closed (Fig. 1d). FFA revealed mild fluorescence leakage beneath the macula (Fig. 1e). The proband also has an affected elder sister aged 36, with over 20 years of progressive vision loss. Both of her eyes underwent vitrectomy for retinal detachment and her visual acuity in both eyes was counting fingers. According to these abnormalities, ARB was suspected, and further genetic testing was performed in this family.

**Fig. 2:**
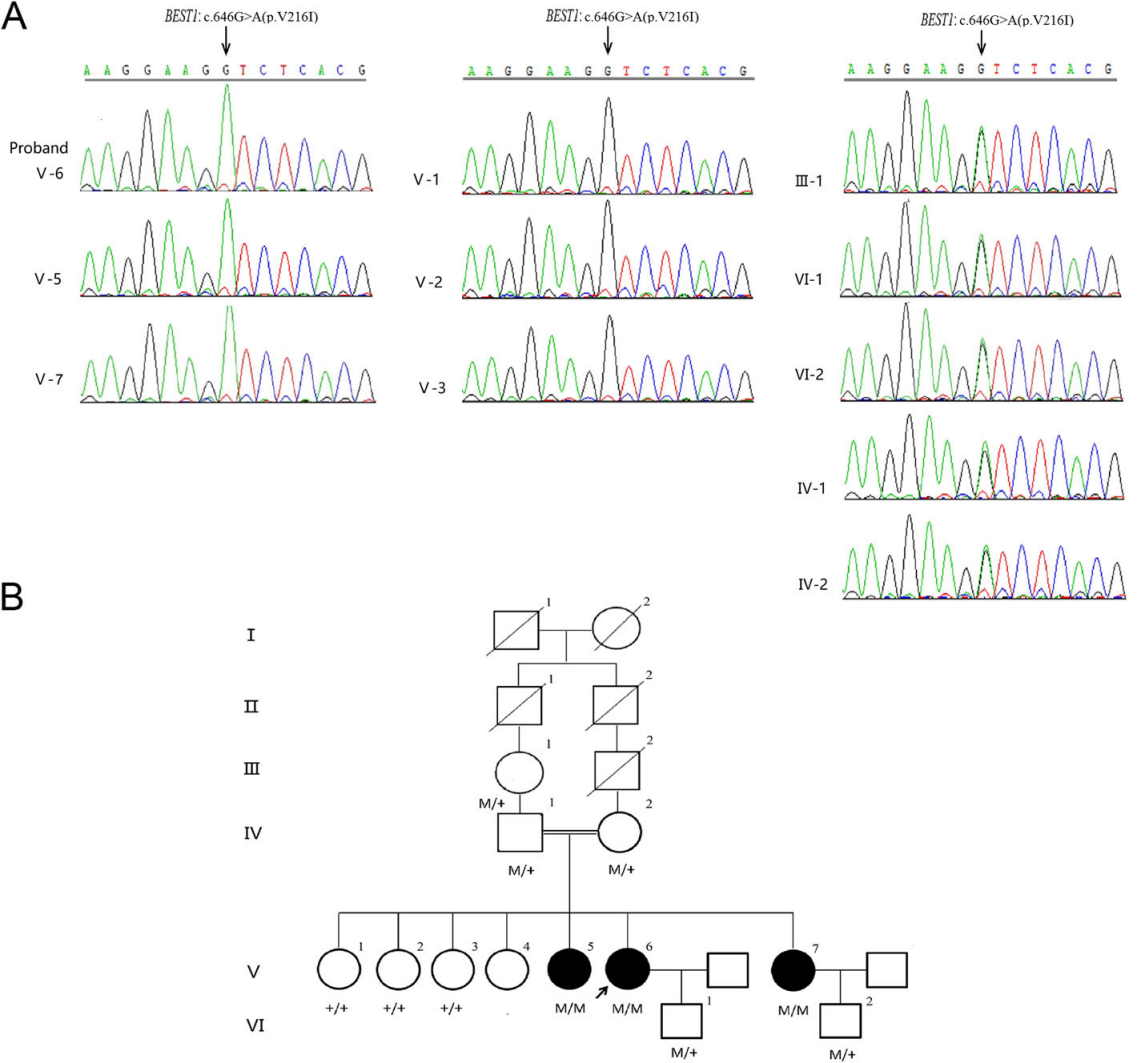
A novel autosomal recessive mutation in the *BEST1* gene c.646G > A (p.V216I) was identified in the family. **a** The results of family separation and analysis showed that the proband (V6), her fifth sister (V5), and her younger sister (V7) carry the homozygous variation. The grandmother (III-1), father (IV1), mother (IV2), and son (VI1) of the proband, her fourth sister (V4), and the son (VI2) of her younger sister carry the heterozygosity. The first sister (V1), the second sister (V2), and the third sister (V3) of the proband carry the wild-type gene. **b** Pedigree. Square symbols denote males, circle symbols denote females, solid symbols indicate the affected, open symbols indicate the unaffected, slash symbols indicate the deceased, an arrow below the symbol indicates the proband, = indicates consanguinity, and + indicates the wild type

**Fig. 3:**
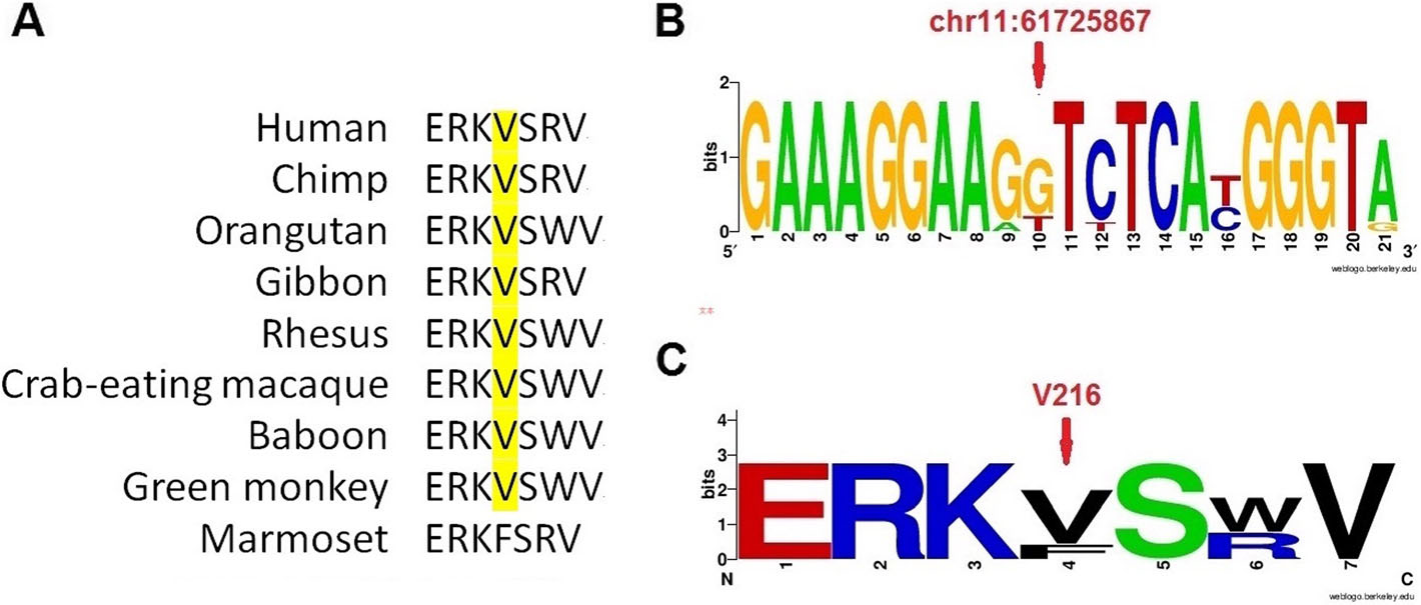
Conservation analysis using the UCSC Genome Browser database. **a** Schematic representation of amino acid sequence alignment around the p.V216I variant in different species. **b** DNA WebLogo of the variant regions. **c** A conservational logo of amino acids of BEST1 protein position 216 (Valine) generated by WebLogo online tool

We performed the target capture sequencing of known retinal disease genes on the proband to identify possible mutation. A homozygous variation in the *BEST1* gene was detected in the 256 gene coding regions associated with retinal diseases in the peripheral blood of the subjects. The variant in the *BEST1* gene (chr11:61725867G > A) was confirmed in the proband. According to the splicing form of the proband (uc010rlu.1), the mutation *BEST1* c.646G > A (p.V216I) was located in exon 7. This is a missense mutation resulting in a replacement of Valine by Isoleucine at amino acid position 216. The G to A allele variant has not been reported in the ExAC database. It was predicted to be deleterious using SIFT (score: 0.017) and FATHMM (score: − 5.32) but not PolyPhen2 (score: 0.007) in dbNSFP (version3.0). All available family members were Sanger sequenced for the segregation analysis (Fig. 2). The results of the family separation and analysis showed that the proband, her fifth sister, and her younger sister were carrying the homozygous variation, and all of them had low vision. The mother and the son of the proband and the son of her younger sister carried the heterozygosity, and the clinical phenotype of the three was normal. The missense mutation was co-segregation across the pedigree and possessed strong amino acid conservation among different species (Fig. 3). Based on the clinical phenotype and genetic variation pattern, the patients were diagnosed with ARB.

**Fig. 4:**
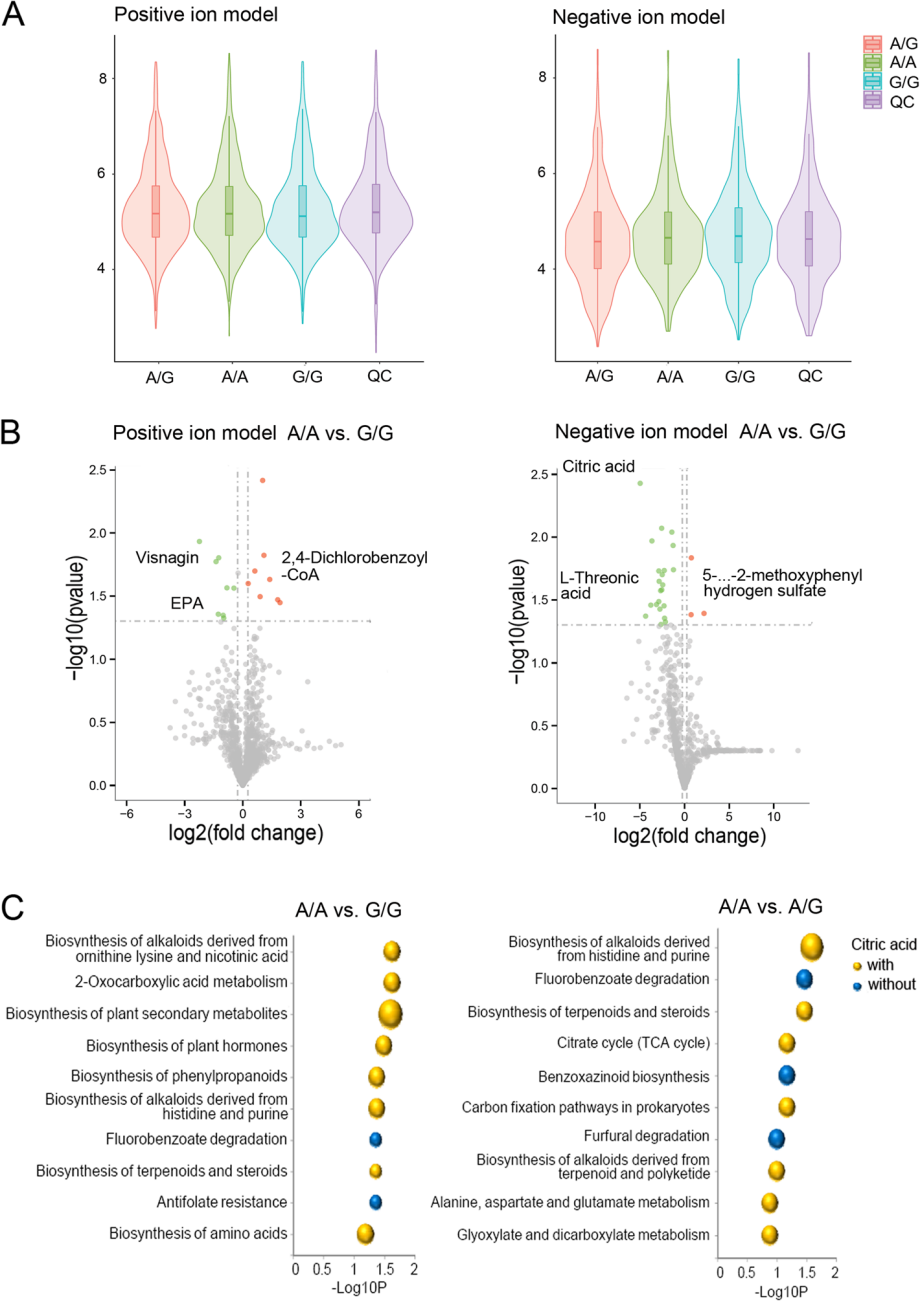
Metabolic profile of ARB patients. **a** Distribution of metabolites in the whole family. **b** The significantly differential expressed metabolites (fold change > 1.2, *P* < 0.05). **c** KEGG einrichment analysis of differential expressed metabolites between ARB patients (A/A, *n* = 2) and their wild-type sisters (G/G, *n* = 3) and between ARB patients (A/A, *n* = 2) and the heterozygosity group (A/G, *n* = 2). The size of the bubble represents the number of genes in each pathway

We performed a metabolomic exploration to evaluate the potential inherited metabolic disorders, which could be diagnostic or therapeutic targets. The distribution of the metabolites is shown in Fig. 4a. We found a very similar blood metabolite profile in this family. Using a criterion (*P* < 0.05, VIP > 1.0), there were 2.0–2.6% (fold change > 1.2) and 1.2–2.0% (fold change > 2.0) metabolites significantly differential expressed between the two groups (Table 1). Compared with her three wild-type sisters (G/G), the proband and her fifth sister, both of whom were of A/A homozygous variant type, showed significantly down-regulated citric acid (fold change > 20), flurothyl, L-Threonic acid, and eicosapentaenoic acid (EPA), etc., as well as up-regulated hydrogen sulfate, manidipine, 2,4-Dichlorobenzoyl-CoA, acetate, etc. (Fig. 4b). Compared with their parents (A/G), they (A/A) also presented significantly down-regulated citric acid and flurothyl, and up-regulated hydrogen sulfate and 2,4-Dichlorobenzoyl-CoA. The enrichment analyses of differential expressed metabolites indicated the central role of citric acid in enriched metabolic pathways (Fig. 4c). Therefore, we recommended lemonade, which is rich in citric acid and vitamin C, for the ARB patients. After three months, OCT of the proband showed a decreased macular edema compared with the initial visit (Additional file 2: Figure S1), although no significant change was found in the FAF.

**Table 1:** Numbers of significantly differential expressed metabolites (DEM) (*P* < 0.05, VIP > 1.0)

Comparison	Total metabolites (n)	DEM (n)	Up-regulated DEM (n)	Down-regulated DEM (n)
Fold change > 1.2				
A_A.vs.G_G_pos	990	16	8	8
A_G.vs.G_G_pos	990	24	17	7
A_A.vs.A_G_pos	990	18	14	4
A_A.vs.G_G_neg	670	25	3	22
A_G.vs.G_G_neg	670	19	6	13
A_A.vs.A_G_neg	670	14	4	10
Fold change > 2.0				
A_A.vs.G_G_pos	990	10	5	5
A_G.vs.G_G_pos	990	15	11	4
A_A.vs.A_G_pos	990	9	5	4
A_A.vs.G_G_neg	670	23	1	22
A_G.vs.G_G_neg	670	5	3	2
A_A.vs.A_G_neg	670	12	3	9

## Discussion

We identified a novel homozygous variation in the *BEST1* gene in a consanguineous family. This was a missense mutation and was co-segregation across the pedigree. In this family, three affected patients with the mutation c.646G > A (p.V216I) were homozygous and had a similar clinical phenotype. Three other members carrying the heterozygosity did not show any phenotype. This finding proved the fact that the inheritance pattern was autosomal recessive. Among betrophinopathies, ARB is presumed to be a retinal disease with a “null” phenotype for Best1 [2, 16]. Based on the clinical abnormalities and gene variation pattern, the patients were diagnosed with ARB. However, EOG was unavailable at the time that patients were examined. That’s a limitation of our workup.

The mutations of *BEST1* can cause various clinical features [1]. To date, over 250 mutations have been explored through the *BEST1* gene, and most mutations occur in the N-terminal part, which is highly conserved among various species [17]. However, the mutation spectrum of the Chinese population may be different from that of patients with other ethnicities. In a Chinese cohort with 20 ARB patients, more than 1/3 mutations (9/22) in the *BEST1* gene were located in exon 7–11, which encoded the C-terminal half of the protein [8]. The other mutations were mainly clustered in the first transmembrane domain and the intracellular regions [8]. In functional prediction analysis, the majority of disease-causing variants were missense mutations [8], which was consistent with our results.

We also performed analyses to assess the association between the missense mutation and disease. In the pathogenicity prediction, SIFT and FATHMM but not PolyPhen2 predicted the mutation to be deleterious. SIFT and PolyPhen2 are the most commonly used online tools for pathogenicity assessment, but they may provide different or even opposite results [18]. Therefore, we further analyzed the potential role of this mutation at the protein level. We found that the amino acid was highly conserved. This may indicate the importance of Valine at amino acid position 216 and support the pathogenicity of this mutation.

Although disease-associated nonsense and frameshift mutations predict a truncated and thus likely inactive protein, the molecular mechanism underlying the effect of missense mutation on protein function and the pathology of disease has not been well elucidated. Uggenti et al. showed that ARB-associated BEST1 protein was degraded via the ubiquitin-proteasome pathway using a stably transfected polarized epithelial cell model [19]. Milenkovic et al. further proved that the ARB-associated missense mutations triggered a strong and fast protein degradation process in the endoplasmic reticulum [20], thereby favoring a decreased stoichiometry of mutant versus normal BEST1 subunits in the assembly of the homo-pentameric BEST1 chloride channel.

In this study, we used a metabolome strategy to investigate a *BEST1* mutation-caused disease. The family members presented very similar metabolic profiles probably because of the same lifestyle, environment, diet, habitat, and genomic background. However, we found a remarkable insufficiency of metabolites, such as citric acid, L-Threonic acid, and EPA, in the blood of the patients compared with that of their sisters. Citric acid or citrate is a key component of the TCA cycle; it serves as a pivotal regulator of intermediary energy metabolism and a chelator for divalent cations, such as like Ca^2+^, Mg^2+^, and Zn^2+^ [21]. It is uptaken by human RPE cells as a preferred nutrient [22], and its buffer is widely used in the in vivo retina experiments. L-Threonate acid is a metabolite of ascorbate (vitamin C), which is highly concentrated in the retina, and plays an important role in physiological function, and protects RPE from oxidant injury [23, 24]. A systemic review including 6150 participants demonstrated that this antioxidant vitamin could slow down the progression of age-related macular degeneration [25]. In addition, the biological importance of Omega-3 fatty acids (EPA and docosahexaenoic acid) in the development of the retina is well established [26]. A recent in vivo study showed that EPA supplementation could reduce lipofuscin granules and slow down the progression of retinal degeneration [27]. Therefore, a lack of these metabolites indicates defective nutrition of the human body, including the retina. According to the results of the metabolome, we recommended a dietary citric acid and vitamin C supplementation in the ARB patients. We found an alleviation of macular edema after a short-term metabolic therapy. The results encouraged the patients to receive longer and more comprehensive nutrient supplementation (e.g., Omega-3 fatty acid) in order to meet their metabolic needs.

This study has limitations. First, the pathogenicity of the mutation was predicted by in silico tools. Further functional experiments are needed to prove the hypothesis. Second, the metabolites in the peripheral plasma may not reflect the changes occurring in the retina. Nevertheless, the present research is a pioneering study that explore the association between altered human metabolism and genetic disease. We believe that some genetic diseases could result in abnormal metabolism and nutritional deficiencies. However, whether nutrient supplementation could ameliorate or even correct the genetic diseases remains to be explored further.

## Conclusion

We provided a new insight into the genomic profiling of the pathological changes in familial ARB. The exploration of the specific metabolism and nutrition status of the genetic disease sheds light on its underlying mechanism and pathophysiology and might provide a potential intervention strategy in the future.

## Supplementary information

**Additional file 1: Table S1.** The list of 256 known retinal disease genes.

**Additional file 2. Figure S1.** The OCT results of the proband.

## Data Availability

The datasets generated and/or analysed during the current study are available in the Google repository, https://drive.google.com/open?id=15KfYv4VKKjw5MysQJxYJhNiK3dTTxHXI, or in the Baidu repository, https://pan.baidu.com/s/1jEXmZZtAMYTwufAEmmCZGw (accession number: ettq).

## Abbreviations

ARB: Autosomal recessive bestrophinopathy
BCVA: Best correct visual acuity
BVMD: Best vitelliform macular dystrophy
CF: Color funduscopy
EPA: Eicosapentaenoic acid
FAF: Fundus autofluorescence
FFA: Fundus fluorescein angiography
InDels: Insertions and Deletions
OCT: Optical coherence tomography
RPE: Retinal pigment epithelium
SNV: Single-nucleotide variants
