# Supplementary material for: Familial autosomal recessive bestrophinopathy: identification of a novel variant in BEST1 gene and the specific metabolomic profile
Source: BMC Med Genet. 2020 Jan 22;21:16. doi: 10.1186/s12881-020-0951-3 (PMC6977271; doi:10.1186/s12881-020-0951-3)
Supplement: Supplementary file 1 — Additional file 1: Table S1. The list of 256 known retinal disease genes. [file 12881_2020_951_MOESM1_ESM.docx]

**Supplementary Data**

**Supplementary Table 1** The list of 256 known retinal disease genes

| ABCA4 | CABP4 | DMD | IFT140 | MVK | PEX7 | RIMS1 | TTC8 |
| --- | --- | --- | --- | --- | --- | --- | --- |
| ABCC6 | CACNA1F | DTHD1 | IFT172 | MYO7A | PGK1 | RLBP1 | TTLL5 |
| ABHD12 | CACNA2D4 | EFEMP1 | IFT27 | NBAS | PHYH | ROM1 | TTPA |
| ACBD5 | CAPN5 | ELOVL4 | IFT81 | NDP | PITPNM3 | RP1 | TUB |
| ADAM9 | CC2D2A | EMC1 | IMPDH1 | NEK2 | PLA2G5 | RP1L1 | TUBGCP4 |
| ADAMTS18 | CDH23 | ERCC6 | IMPG1 | NEUROD1 | PLA2G6 | RP2 | TUBGCP6 |
| ADGRV1 | CDH3 | EXOSC2 | IMPG2 | NMNAT1 | PLK4 | RP9 | TULP1 |
| ADIPOR1 | CDHR1 | EYS | INPP5E | NPHP1 | PNPLA6 | RPE65 | UNC119 |
| AGBL5 | CEP164 | FAM161A | INVS | NPHP3 | POC1B | RPGR | USH1C |
| AHI1 | CEP250 | FBLN5 | IQCB1 | NPHP4 | POMGNT1 | RPGRIP1 | USH1G |
| AIPL1 | CEP290 | FBN1 | ITM2B | NR2E3 | PRCD | RPGRIP1L | USH2A |
| ALMS1 | CERKL | FLVCR1 | JAG1 | NR2F1 | PRDM13 | RS1 | VCAN |
| APOB | CFH | FRMD7 | KCNJ13 | NRL | PROM1 | RTN4IP1 | WDPCP |
| ARL2BP | CHM | FSCN2 | KCNV2 | NYX | PRPF3 | SAG | WDR19 |
| ARL3 | CIB2 | FZD4 | KIAA1549 | OAT | PRPF31 | SDCCAG8 | WFS1 |
| ARL6 | CLN3 | GDF6 | KIF11 | OFD1 | PRPF4 | SEMA4A | ZNF408 |
| ATF6 | CLRN1 | GNAT1 | KIZ | OPA1 | PRPF6 | SLC24A1 | ZNF423 |
| ATXN7 | CLUAP1 | GNAT2 | KLHL7 | OPA3 | PRPF8 | SLC25A46 | ZNF513 |
| BBIP1 | CNGA1 | GNB3 | LAMA1 | OPN1LW | PRPH2 | SLC7A14 |  |
| BBS1 | CNGA3 | GNPTG | LCA5 | OPN1MW | PRPS1 | SNRNP200 |  |
| BBS10 | CNGB1 | GPR125 | LRAT | OPN1SW | RAB28 | SPATA7 |  |
| BBS12 | CNGB3 | GPR143 | LRIT3 | OTX2 | RAX2 | SPP2 |  |
| BBS2 | CNNM4 | GPR179 | LRP5 | PANK2 | RB1 | TEAD1 |  |
| BBS4 | COL11A1 | GRK1 | LZTFL1 | PAX2 | RBP3 | TIMM8A |  |
| BBS5 | COL2A1 | GRM6 | MAK | PCDH15 | RBP4 | TIMP3 |  |
| BBS7 | COL9A1 | GUCA1A | MAPKAPK3 | PCYT1A | RD3 | TMEM126A |  |
| BBS9 | CRB1 | GUCA1B | MERTK | PDE6A | RDH11 | TMEM216 |  |
| BEST1 | CRX | GUCY2D | MFN2 | PDE6B | RDH12 | TMEM237 |  |
| C10orf11 | CSPP1 | HARS | MFRP | PDE6C | RDH5 | TOPORS |  |
| C12orf65 | CWC27 | HGSNAT | MFSD8 | PDE6G | REEP6 | TREX1 |  |
| C1QTNF5 | CYP4V2 | HK1 | MIR204 | PDE6H | RGR | TRIM32 |  |
| C21orf2 | DFNB31 | HMCN1 | MKKS | PDZD7 | RGS9 | TRNT1 |  |
| C2orf71 | DHDDS | HMX1 | MKS1 | PEX1 | RGS9BP | TRPM1 |  |
| C8orf37 | DHX38 | IDH3B | MTTP | PEX2 | RHO | TSPAN12 |  |
