# Supplementary figures and images for: Familial autosomal recessive bestrophinopathy: identification of a novel variant in BEST1 gene and the specific metabolomic profile
Source: BMC Med Genet. 2020 Jan 22;21:16. doi: 10.1186/s12881-020-0951-3 (PMC6977271; doi:10.1186/s12881-020-0951-3)

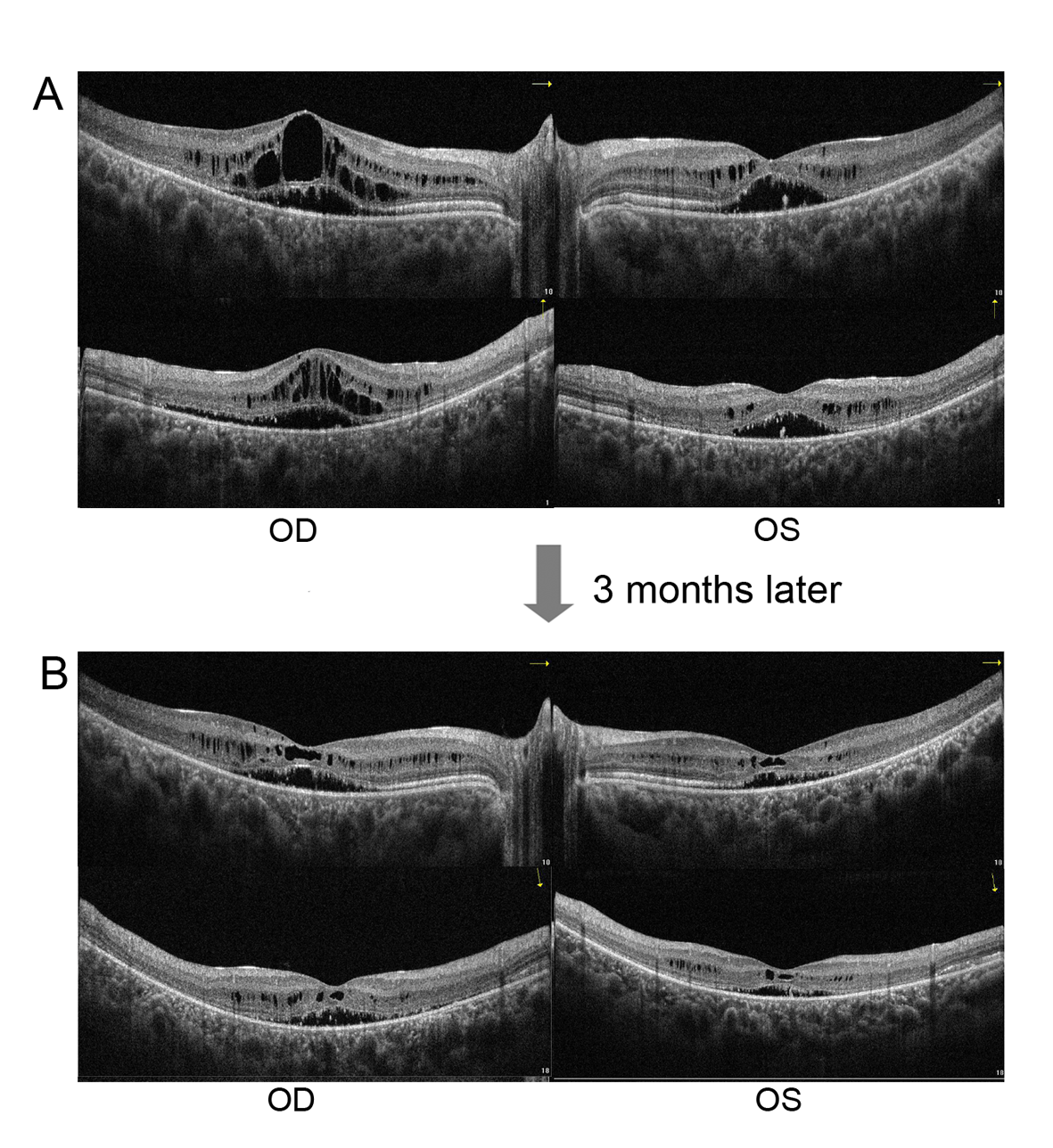

Supplement: Supplementary file 2 — Additional file 2. Figure S1. The OCT results of the proband. [file 12881_2020_951_MOESM2_ESM.tif]
